# Bidirectional Electromagnetically Induced Transparency Based on Coupling of Magnetic Dipole Modes in Amorphous Silicon Metasurface

**DOI:** 10.3390/nano11061550

**Published:** 2021-06-11

**Authors:** Shuang Liu, Jingxin Dong, Jiangnan Si, Weiji Yang, Xuanyi Yu, Jialin Zhang, Xiaoxu Deng

**Affiliations:** 1 State Key Laboratory of Advanced Optical Communication Systems and Networks, Key Laboratory for Laser Plasmas (Ministry of Education), School of Physics and Astronomy, Shanghai Jiao Tong University, Shanghai 200240, China; 503313461@sjtu.edu.cn (S.L.); 1359231351370@sjtu.edu.cn (J.S.); yangweiji@sjtu.edu.cn (W.Y.); yxy1593725@sjtu.edu.cn (X.Y.); dorismalfoy@sjtu.edu.cn (J.Z.); 2 School of Electronic Information and Electrical Engineering, Shanghai Jiao Tong University, Shanghai 200240, China; jx_dong@sjtu.edu.cn

**Keywords:** electromagnetically induced transparency, magnetic dipole modes, a-Si metasurface

## Abstract

A bidirectional electromagnetically induced transparency (EIT) arising from coupling of magnetic dipole modes is demonstrated numerically and experimentally based on nanoscale a-Si cuboid-bar metasurface. Analyzed by the finite-difference time-domain (FDTD) Solutions, both the bright and dark magnetic dipole mode is excited in the cuboid, while only the dark magnetic dipole mode is excited in the bar. By breaking the symmetry of the cuboid-bar structure, the destructive interference between bright and dark magnetic dipole modes is induced, resulting in the bidirectional EIT phenomenon. The position and amplitude of simulated EIT peak is adjusted by the vertical spacing and horizontal spacing. The EIT metasurface was fabricated by Electron-Beam Lithography and deep silicon etching technique on the a-Si film deposited by Plasma-Enhanced Chemical Vapor Deposition. Measured by a convergent spectrometer, the fabricated sample achieved a bidirectional EIT peak with transmission up to 65% and 63% under forward and backward incidence, respectively. Due to the enhanced magnetic field induced by the magnetic dipole resonance, the fabricated bidirectional EIT metasurface provides a potential way for magnetic sensing and magnetic nonlinearity.

## 1. Introduction

Electromagnetically induced transparency (EIT) has attracted enormous interest due to its unique characteristics, including extreme dispersion and enhanced nonlinear effect. So far, an EIT phenomenon has been reported in various systems and structures, among which three-level atomic system [1,2,3,4,5], coupled waveguide [6,7,8,9,10,11,12,13], and metamaterials [14,15] are typical examples. EIT metamaterials require a proper arrangement of geometry structure, of which experimental condition is simple compared with that needed in atomic EIT system. EIT in plasmonic metamaterials has covered a broad range of wavelength from visible to microwave region [16,17,18,19,20] with silver and gold as the chief materials. Graphene-based metamaterials have been proposed to induce tunable EIT effect by adjusting the applied bias on the graphene [21,22,23,24,25,26], which serve as building blocks for adjustable optical devices. EIT dielectric metamaterials based on Mie-resonance, where the displacement current replaces the conduction current, feature greatly reduced non-radiative loss compared with plasmonic counterparts, providing a promising platform for application in low-loss, slow-light devices [27,28,29], high-efficiency optical sensors [30,31], polarization convertors [32], and high-modulation optical switches [33,34].

EIT in dielectric metasurfaces has been widely investigated, which originates from the destructive interference between various modes of Mie-resonance, including coupling of electric-electric dipole, electric-magnetic dipole, and electric dipole-electric quadrupole. Coupled electric-electric dipole induced transparency has been reported in silicon cross nanostructure metasurface [35], which realizes optical refractive sensing with a figure-of-merit of 42 and slow-light effect with a group delay of 0.65 ps. Coupled electric-magnetic dipole induced transparency has been verified experimentally in silicon ring-bar nanostructure metasurface [36] and disk-bar nanostructure metasurface [37], respectively. The former achieves optical refractive index sensing with a figure-of-merit of 103 and the later generates high-harmonic signal with orders of magnitude higher compared to an unpatterned Si film. Silicon dolmen-like nanostructure metasurface has been employed for realization of coupled electric dipole-electric quadrupole induced transparency [38], of which Q-factor reaches 2680 and group refractive index exceeds 200. However, few works have focused on the EIT behavior which results from the magnetic-magnetic dipole coupling in dielectric metasurfaces.

In this paper, a bidirectional EIT induced by coupling between magnetic dipoles is analyzed numerically and experimentally in nanoscale a-Si cuboid-bar metasurface. Based on discrete dipole approximation (DDA) theory, the magnetic dipoles in both cuboid and bar nanostructure, treated as bright and dark mode, are calculated from the simulated displacement currents, which interfere destructively to generate EIT phenomenon. The impact of spacing between cuboid and bar on the EIT peak is investigated by numerical simulations, and the results indicate that the EIT peak is tuned by the coupling separations and structural asymmetry, which is the spacing between cuboid and bar in different direction, respectively, arising from the shift of the dark mode and change of coupling effect. The bidirectional EIT metasurface was fabricated by Electron-Beam Lithography (EBL) and deep silicon etching technique. The transmission spectra of the fabricated EIT metasurface are measured by a convergent spectrometer, which exhibit a bidirectional EIT peak with transmission up to 65% and 63% under forward and backward incidence, respectively. Due to the enhanced magnetic field induced by the magnetic dipole Mie resonance inside the cuboid and bar nanostructure and near-zero nonradiative loss of a-Si, the proposed bidirectional EIT metasurface which realize EIT phenomenon under bidirectional incidence, has potential applications in the field of magnetic sensing, magnetic nonlinearity and bidirectional devices.

## 2. Materials and Methods

**Figure 1:**
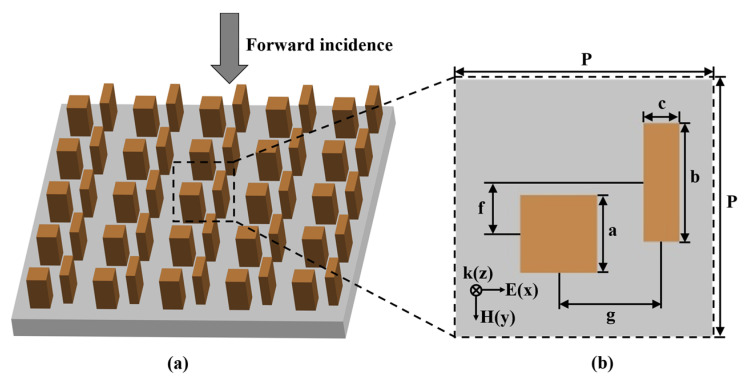
(**a**) Schematic of the near-infrared bidirectional EIT metasurface based on a-Si cuboid-bar nanostructure periodic arrays on the SiO_2_ substrate. The black dash box shows a unit cell. (**b**) Top view of the unit cell.

The schematic of the near-infrared bidirectional EIT metasurface is shown in Figure 1a, which is an a-Si cuboid-bar nanostructure periodic array on the SiO_2_ substrate. The unit cell of the periodic array, which comprises a cuboid resonator and a bar resonator, is shown in Figure 1b. The side length of the cuboid is a = 150 nm. The length and width of the bar is b = 230 nm and c = 70 nm, respectively. The height of the two resonators is h = 270 nm. The center distance between two resonators in x and y direction (along the short and long axis of the bar) is g and f, respectively. The period of unit cell in x and y direction is P = 500 nm. The refractive index of SiO_2_ is 1.528.

Transmission spectra and displacement currents of the metasurfaces are simulated by finite-difference time-domain (FDTD) Solutions. In the simulations, a semi-infinite substrate is added below the metasurfaces, and a monitor in the substrate is set 500 nm below the metasurfaces to calculate the transmission from the ratio of the power flowing into the monitor to the power of light source. x component and y component of magnetic dipole moments inside the cuboid and the bar of the bidirectional EIT metasurface are calculated based on discrete dipole approximation (DDA) theory [39,40], which is given by:

$$m_{1}=m_{ycuboid}=\frac{1}{2}\int_{cuboid}^{\;}zJ_{x}-xJ_{z}d^{3}r$$

$$m_{2}=m_{xbar}=\frac{1}{2}\int_{bar}^{\;}yJ_{z}-zJ_{y}d^{3}r$$

$$m_{3}=m_{xcuboid}=\frac{1}{2}\int_{cuboid}^{\;}yJ_{z}-zJ_{y}d^{3}r$$

where $J_{x}$, $J_{y}$ and $J_{z}$ is the x, y and z component of displacement current, respectively. The discrete dipole approximation decomposes the cuboid and bar nanostructures into multiple point dipoles, each of which generates a displacement current which is used to calculate the magnetic dipole moments.

The correlation coefficient is introduced to evaluate the matching between the numerical and experimental EIT peaks, which is given by:

$$r\left(T_{sim},T_{exp}\right)=\frac{\sum \left(T_{sim}-{\bar{T}}_{sim}\right)\left(T_{exp}-{\bar{T}}_{exp}\right)}{\sqrt{\sum {\left(T_{sim}-{\bar{T}}_{sim}\right)}^{2}{\left(T_{exp}-{\bar{T}}_{exp}\right)}^{2}}},$$

where $T_{sim},\;T_{exp}$ is the simulated and experimental transmissions of the EIT peak, respectively, and ${\bar{T}}_{sim},\;{\bar{T}}_{exp}$ is the average simulated and experimental transmission of the EIT peak.

**Figure 2:**
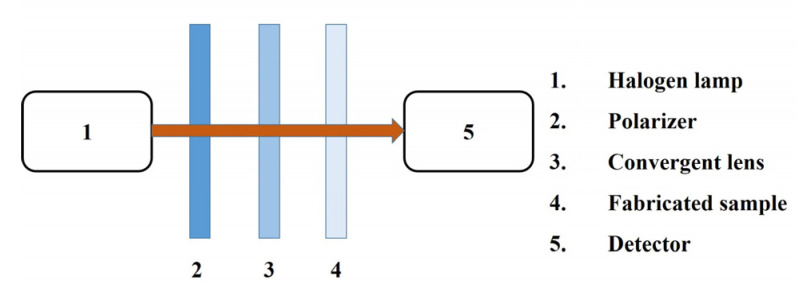
The schematic presentation of the convergent spectrometer with a polarizer.

An a-Si film is grown on the SiO_2_ substrate by Plasma-Enhanced Chemical Vapor Deposition (PECVD). The thickness of the SiO_2_ substrate is 1 mm. The cuboid-bar nanostructure EIT metasurface is fabricated by electron-beam lithography and deep silicon etching technique. The transmission spectra of the fabricated cuboid-bar nanostructure EIT metasurface are measured by a convergent spectrometer. As shown by Figure 2, a polarizer is added in the convergent spectrometer to ensure that the incident light is polarized along the short axis of the bar in the bidirectional EIT metasurface.

## 3. Results and Discussion

### 3.1. Numerical and Simulated Results

**Figure 3:**
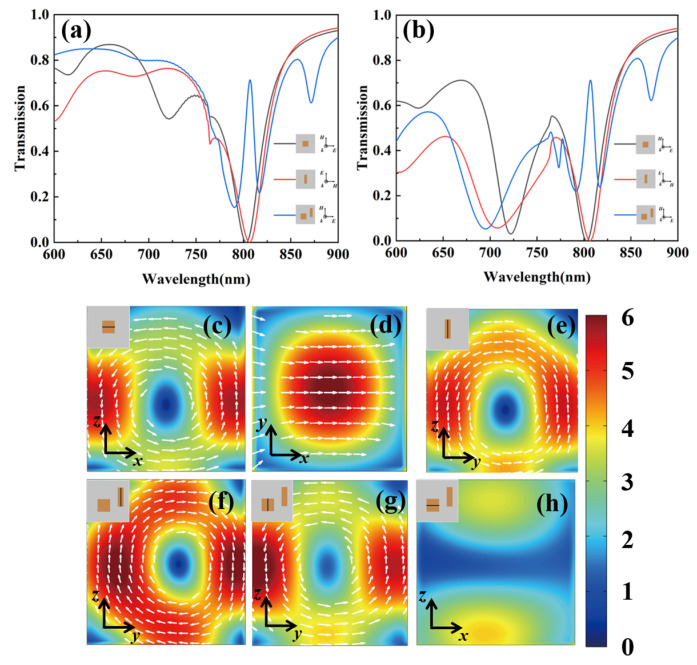
Simulated transmission spectrum for sole-cuboid nanostructure metasurface, sole-bar nanostructure metasurface and bidirectional EIT metasurface with f = 100 nm and g = 200 nm under forward incidence (**a**) and backward incidence (**b**). Distribution of electric field in x-z plane inside the cuboid of the sole-cuboid nanostructure metasurface at 803 nm (**c**), in x-y plane inside the cuboid of the sole-cuboid nanostructure metasurface at 722 nm (**d**), in y-z plane inside the bar of the sole-bar nanostructure metasurface at 806 nm (**e**). Distribution of electric field in y-z plane inside the bar (**f**), in y-z plane inside the cuboid (**g**), and in x-z plane inside the cuboid (**h**) of the near-infrared bidirectional EIT metasurface at the EIT peak.

Transmission spectra for sole-cuboid nanostructure metasurface, sole-bar nanostructure metasurface and bidirectional EIT metasurface are simulated by FDTD Solutions. The simulated transmission spectrum of the sole-cuboid nanostructure metasurface with the incident electric field E along *x* axis exhibits a dip at 803 and at 722 nm under backward incidence, as shown by black curve in Figure 3b. At the transmission dip 803 nm, the enhanced circular electric field in the cuboid simulated by FDTD at the x-z plane corresponds to magnetic dipole resonance oriented in y direction, which forms the bright mode shown in Figure 3c. At the transmission dip 722 nm, the enhanced linear electric field in the cuboid simulated by FDTD at the x-y plane corresponds to electric dipole resonance oriented in x direction shown in Figure 3d. Under forward incidence, the dip at 803 nm remains unchangeable while the dip at 722 nm reduces significantly in the simulated transmission spectrum of sole-cuboid nanostructure metasurface shown by the black curve in Figure 3a, indicating that the magnetic dipole mode is bidirectional but the electric dipole mode is not. The scattering electric field induced by magnetic dipole is related to the permeability, which remains unchangeable under bidirectional incidence as the permeability is constant to 1 for both a-silicon metasurfaces and substrate. While, the scattering electric field induced by electric dipole is related to the permittivity, which changes with the direction of incidence as the permittivity distribution is different under forward incidence (incident from air side) and backward incidence (incident from substrate side). Due to the C_4_ symmetry of the cuboid, magnetic dipole resonance oriented in x direction is also excited by the E along *y* axis at 803 nm and cannot be excited by the x-polarized light directly, which serves as the dark mode under x-polarized incidence. In the simulated transmission spectrum of the sole-bar nanostructure metasurface with the incident electric field E along y axis under bidirectional incidence, a pronounced dip, as shown by red curve in Figure 3a,b, occurs at 806 nm, around which circular electric field at the y-z plane of the bar is simulated in Figure 3e, so a bidirectional magnetic dipole resonance in x direction forms and serves as the dark mode under x-polarized incidence. A transparency window centered at 809 nm is observed in the simulated transmission spectrum of the bidirectional EIT metasurface with the incident electric field E along x axis under bidirectional incidence, as shown by blue curve in Figure 3a,b. The simulated electric field distribution at the EIT peak inside the bar resonator and the cuboid resonator at the y-z plane both exhibit an enhanced loop, as shown in Figure 3f,g, respectively, whereas the circular electric field inside the cuboid resonator at the x-z plane vanishes, as shown in Figure 3h. Hence, the dark modes not only in the bar resonator but also in the cuboid resonator are excited simultaneously by the bright mode other than external electric field. When the structural asymmetry is introduced to the metasurface, the bright bidirectional magnetic dipole (MD) mode and dark bidirectional MD mode, arranged in close proximity in the special and frequency domain, interfere with each other destructively, suppressing the bright mode and consequently leading to the bidirectional EIT phenomenon.

**Figure 4:**
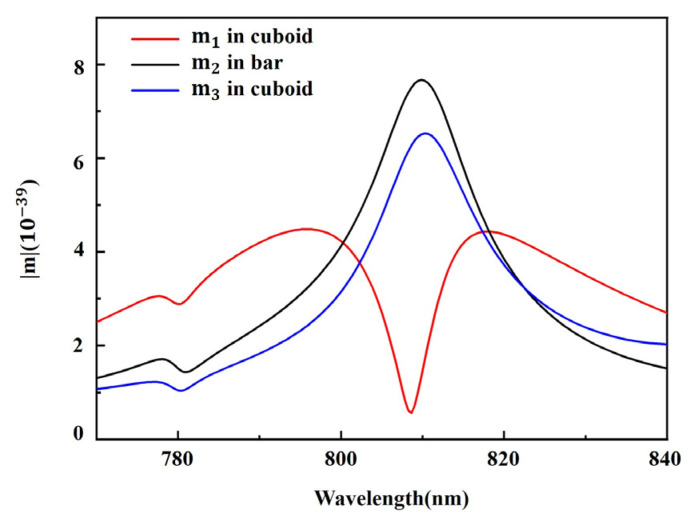
Calculated magnetic dipole moment inside the cuboid and the bar of the bidirectional EIT metasurface.

The magnetic dipole moments in the bidirectional EIT metasurface are calculated from the displacement currents simulated by FDTD. The magnetic dipole moment $m_{1}$ in cuboid (x component of the magnetic dipole), which relates to bright mode, reaches minimum at 809 nm, shown by red curve in Figure 4, whereas the magnetic dipole moments $m_{2}$ in bar and $m_{3}$ in cuboid (y component of the magnetic dipole), which relate to dark mode, both exhibit a peak at 810 nm, as shown by black curve and blue curve in Figure 4, respectively. The maximum of the magnetic dipole moments $m_{2}$ and $m_{3}$ corresponds to the minimum of the magnetic dipole moment $m_{1}$, that is, the enhancement of the dark mode is accompanied by the decrease of the bright mode. The electromagnetic energy is transferred from the bright mode $m_{1}$ into the dark modes $m_{2}$ and $m_{3}$ due to the near-field coupling, resulting in the dip of $m_{1}$ and the peak of $m_{2}$ and $m_{3}$. The destructive interference between bright and dark modes results in the EIT phenomenon in cuboid-bar metasurface.

**Figure 5:**
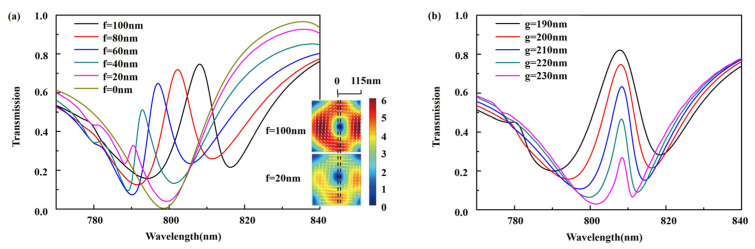
Simulated transmission spectrum of the periodic cuboid-bar nanostructure EIT metasurface with (**a**) different vertical spacing, (**b**) different horizontal spacing g between the cuboid and the bar. Inset shows the distribution of electric field with displacement currents in y-z plane inside the bar when f = 20 nm and f = 100 nm at the EIT peak.

The transmission spectra of the cuboid-bar nanostructure EIT metasurface are simulated by FDTD Solutions with vertical spacing f and horizontal spacing g. The simulated transmission spectra of EIT metasurface with different vertical spacing f are shown in Figure 5a, where the EIT peak increases and red-shifts with vertical spacing f varying from 0 to 100 nm. When f equals to 0, the EIT phenomenon vanishes and only the MD resonance dip remains in the simulated transmission spectrum. Increasing structural asymmetry f will enhance the coupling effect between the cuboid and bar, and change the spatial location of the center of the circular displacement currents inside the bar, shown by inset in Figure 5a, resulting in red-shift of the dark modes and consequently shift and enhancement of the EIT peak. The simulated transmission spectra of EIT metasurface with horizontal spacing g are shown in Figure 5b. When the horizontal spacing g increases from 190 to 230 nm, the EIT peak increases and broadens with the spectral position fixed at 809 nm as a result of enhancement of coupling among the magnetic dipoles in cuboid and bar.

### 3.2. Experimental Results

**Figure 6:**
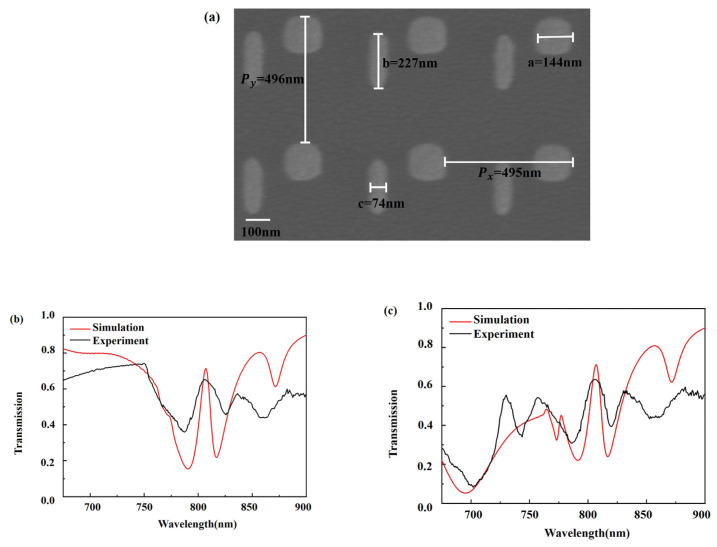
(**a**) SEM image of the fabricated bidirectional EIT metasurface. The measured and simulated transmission spectrum of the bidirectional EIT metasurface (**b**) under forward incidence, (**c**) under backward incidence.

Scanning electron microscopy (SEM) image of the fabricated sample is shown in Figure 6a. The size of the cuboid and bar is close to 150 nm × 150 nm, 230 nm × 70 nm, respectively. Period of the cuboid-bar array nanostructure is P_x_ ≈ 500 nm, P_y_ ≈ 500 nm. The total area of the fabricated sample is 300 μm × 300 μm. The measured transmission spectra of the fabricated EIT metasurface exhibit a bidirectional EIT peak with a maximum of 65% and a maximum of 63% at 806 nm under forward and backward incidence, respectively, shown by black curve in Figure 6b,c. The experimental spectra are in the same trend with the simulated results shown by red curve in Figure 6b,c, despite that the EIT peak in the measured spectra is reduced and broadened, which arises from the imperfection of the fabricated EIT metasurfaces and the formation of a low refractive index oxide layer. The correlation coefficient between the experimental and simulated EIT peak reaches 0.73 and 0.78 under forward and backward incidence, respectively, which demonstrates the good matching between the measured and simulated EIT peak.

## 4. Conclusions

In conclusion, a nanoscale a-Silicon cuboid-bar metasurface is investigated numerically and demonstrated experimentally to achieve bidirectional EIT response induced by the near field coupling between magnetic dipole modes. The cuboid supports both bright and dark magnetic dipole mode, while the bar only supports dark magnetic dipole mode. When the structural symmetry of metasurface is broken, EIT stems from the destructive interference between bright and dark magnetic dipole modes. Simulated by FDTD Solutions, the EIT peak shifts and fluctuates according to the vertical spacing (structural asymmetry) and horizontal spacing between cuboid and bar, which is caused by the relocation of the center of the displacement currents and change of coupling effect. A convergent spectrometer is utilized to measure the transmission spectra of the EIT metasurface which is fabricated by Electron-Beam Lithography (EBL) and deep silicon etching technique. The EIT peak in experiments arrives 65% and 63% for light incident from front and back side, respectively, which exhibits the same trend as the simulations. Due to the enhanced magnetic field induced by the magnetic dipole Mie resonance and near-zero nonradiative loss of a-Si, the proposed bidirectional EIT phenomenon is prospective in the field of magnetic sensing and magnetic nonlinearity.

## Data Availability

The data presented in this study are available on request from the corresponding author.
